# Tumor growth inhibitory activity of the P2X7 receptor antagonist AZ10606120 in two cell lines of human glioblastoma

**DOI:** 10.1007/s11302-026-10144-8

**Published:** 2026-03-28

**Authors:** Michela Graziano, Silvia Bono, Michela Pizzoferrato, Chiara Ferraro, Flavia Grossi, Pierluigi Navarra, Maria Martire, Lucia Lisi

**Affiliations:** Section of Pharmacology, Department of Translational Medicine and Surgery, Catholic University, Medical School, Largo F. Vito 1, 00168 Rome, Italy

**Keywords:** Glioblastoma, Purinergic system, P2X7 receptor, AZ10606120, U87MG, T98G cell lines

## Abstract

Glioblastoma multiforme (GBM) is among the most aggressive and lethal primary brain tumors. P2X7 is a purinergic receptor overexpressed in GBM, whose activation can promote tumor growth. Two major splice variants of the P2X7 receptor, isoforms A and B, are expressed in GBM cells. In our study, both isoforms A and B were present in GBM cells, with a slight prevalence of isoform B expression in the U87MG cell line compared to the T98G cell line. In GBM cell cultures, we evaluated the effects of P2X7 receptor activation/inhibition on cell growth. Exposure of GBM cells to various concentrations of ATP did not alter cellular activity, whereas inhibition of the P2X7 receptor with the antagonist AZ10606120, but not with A740003, significantly reduced GBM cell viability and proliferation. The U87MG cell line, which expresses the B isoform more highly, was more sensitive to the antiproliferative effects of AZ10606120. To clarify the mechanisms of AZ10606120-induced tumor inhibition, we measured the expression of proteins involved in cell repair and survival processes. AZ10606120 induced an increase in caspase-3 and p21 expression, demonstrating that P2X7R blockade can induce tumor cell death, likely by apoptosis. AZ10606120 also stimulated interleukin-6 (IL-6) release in GBM cells and induced phosphorylation of CREB and STAT3. Blockade of P2X7R by AZ10606120 appears to activate a pro-tumor IL-6/STAT3 axis. Increased IL-6 secretion and activation of the CREB and STAT3 pathways are negative prognostic signs that highlight the need to combine P2X7R inhibition with AZ10606120 with other therapies.

## Introduction

Glioblastoma multiforme (GBM) is one of the most lethal brain tumors, with a median survival rate of approximately 15 months [1]. Despite extensive surgical removal and intensive follow-up treatment, nearly all GBM tumors recur after therapy. The standard therapeutic approach for GBM involves surgery, followed by radiotherapy and chemotherapy (with temozolomide-TMZ, an alkylating agent) for 6 weeks and then six additional cycles of TMZ maintenance [2]. GBM recurrence is due to its highly infiltrative nature, the high heterogeneity of intratumoral and intertumoral cells that characterize it, and the difficulty of drug diffusion across the blood–brain barrier [3, 4].

Purinergic receptors are known to be expressed by malignant tumors of various origins, by tumor-infiltrating inflammatory cells and by supporting stromal cells, for these reasons purinergic receptors can influence tumor growth in various ways [5, 6]. Among purinergic receptors, the P2X7 receptor (P2X7R) is overexpressed in GBM [7, 8]. P2X7R is present in all tissues and organs under physiological conditions and its expression changes in a wide range of tumors [9]. The active role of P2X7R in tumor progression makes this receptor an interesting target for the development of new therapies and in particular P2X7R antagonist drugs seem to be promising for the treatment of GBM [10, 11].

Adenosine triphosphate (ATP) is the major agonist of purinergic P2X receptors, trimeric channels that regulate the transmembrane movement of cations [12]. ATP functions both as a rapid neurotransmitter and as a modulator, acting directly or through its conversion to adenosine diphosphate (ADP) and adenosine. ATP is a widespread regulatory signal that acts by activating both G protein-coupled receptors (P2Y) and channel receptors (P2X) [13], thus it can influence cellular activity in various ways in different types of cancer. ATP and its derivatives stimulate GBM invasion by modifying the local microenvironment to favor tumor progression [14]. P2X7R receptors can be distinguished from other P2X receptors by their low affinity for ATP; ATP shows a physiological EC_50_ towards P2X7R receptors of 0.3–1 mM [15]. Under conditions of continuous activation or exposure to high concentrations of ATP or agonists [16], the configuration of P2X7R changes and the channel forms a large pore in the membrane that allows the passage of large water-soluble molecules up to 900 Da [17].

P2X7R can consist of several isoforms; at least nine splice variants of the human P2X7 receptor, designated P2X7A–J, have been identified, where P2X7A is the well-characterized full-length P2X7 receptor [18]. Of the 9 splice variants, 4, P2X7B, P2X7E, P2X7G and P2X7J, lack the C-terminal segment. Among these, the P2X7B isoform can form a functional channel in the plasma membrane [19]. P2X7B is of particular interest due to its broad tissue expression, sequence similarity to other P2X receptors and the loss of cytotoxic activity typical of P2X7R [20]. The presence of a shorter P2X7R isoform, devoid of cytotoxic activity, suggests that P2X7R responses can be finely regulated by the expression of its various isoforms, with obvious implications for the control of cell growth. Furthermore, P2X7B can co-assemble with the isoform P2X7A to form a P2X7A + B heterotrimer leading to variations in the original functions of P2X7R. In some studies, it has been observed that the P2X7A variant is able to produce antitumor effects that promote cell death, while the P2X7B variant seems to be implicated in the trophic, metastatic and resistance properties of tumor cells [21].

In this study, we measured the expression of isoforms A and B in two GBM cell lines, U87MG and T98G. After characterizing the P2X7R isoforms present in our cell cultures, we analyzed the effects of P2X7R activation and inhibition on cell growth. The ATP agonist and antagonist A740003 showed no effect on cell growth in U87MG and T98G GBM cultures, while the antagonist AZ10606120 was shown to significantly inhibit tumor growth in GBM cells. AZ10606120 is a negative allosteric inhibitor that binds P2X7R with an IC_50_ of approximately 10 nM [22] and has been shown to inhibit tumor growth of GBM cell lines unlike other antagonists such as brilliant blue G (BBG) or oxidized ATP (o-ATP) probably due to its high potency and selectivity [23].

Significant progress has been made in understanding the potential connection between P2X7R and cancer; however, the specific mechanisms through which P2X7R participates in tumorigenesis are not fully elucidated. Many studies indicate that P2X7R plays a key role in tumor proliferation, tumor cell migration, and the expression of inflammation-promoting factors in various human glioma cell lines [24]. In the present study, we analyzed the cellular mechanisms underlying the tumor growth inhibitory effect induced by AZ10606120. From the obtained data, it appears that AZ10606120 effectively inhibits tumor proliferation by activating apoptotic death mechanisms.

In addition to inhibiting cell growth, initial exposure to AZ10606120 triggers the release of interleukin 6 (IL-6). IL-6 is an important cytokine for the activation of pro-oncogenic signaling pathways in GBM [25]. In cells exposed to AZ10606120, we measured increased expression of STAT3 and CREB. Activation of these IL-6-activated signaling pathways may be a temporary rescue response mounted by cells damaged by P2X7R silencing.

## Materials and methods

### Cell cultures

T98G is a fibroblast-like cell line that was isolated from the brain of a glioblastoma multiforme white, 61-year-old, male patient. U87MG is a cell line with epithelial morphology that was isolated from malignant gliomas (glioblastoma) from a male patient. Both cell lines were cultured in Dulbecco’s modified Eagle’s medium–high glucose (DMEM) (Sigma Cat no. D6429-500 mL) supplemented with 10% FBS (fetal bovine serum), 1% penicillin–streptomycin at 37 °C in a 5% CO2 environment. When cells reached 80% of confluence, they were split and sub-cultured at a concentration of 10.000 cells/well in 96-well plates for experimental procedures.

### Cell treatments

U87MG and T98 cells were treated for 72 h with ATP (ATP disodium salt by Tocris Biosciences Catalog #3245) that was dissolved in media and the pH was adjusted to 7.4 with NaOH, and with the following P2X7R antagonists: AZ10606120 (Tocris Biosciences Catalog #3323) and A74003 (Tocris Biosciences Catalog #3701).

### Viability assay

XTT (TACS® XTT Cell Proliferation Assay Kit, R&D System, Minennapolis, MN, Cat. No. 4891–025-K) was performed, according to the manufacturer’s procedure. U87MG and T98G were plated in a 96-well plate at a density of 10,000 cells/well. XTT assay was performed at 24 h, 48 h, 72 h. Cell viability was examined by measuring the reduction of formazan salts (3-(4,5-dimethylthiazole-2-yl)−5-(4-sulfophenyl)−2H-tetrazolium MTS) contained in the kit. Living cells reduce MTS to purple formazan salts, which absorb light at 492 nm. Cell viability was assessed by measuring absorbance using a microplate photometer (Victor 4, PerkinElmer, Waltham, MA, USA) and was expressed as the percentage of cell viability in relation to the untreated controls. The absorbance values measured with a plate spectrophotometer are directly proportional to cell viability.

### Toxicity assay

LDH assay (CytoTox 96® Non-Radioactive Cytotoxicity Assay, Promega, Madison, WI, USA, Cat. No.: G1780) was performed, following manufacturer’s instructions. U87MG and T98G were plated and treated with the same procedure used for XTT assay and extracellular and intracellular LDH amount was read by measuring absorbance at 490 nm with a microplate photometer (Victor 4, PerkinElmer, Waltham, MA, USA). LDH assay was performed at 24 h, 48 h, 72 h. Extracellular LDH was measured in the medium released from cells, while intracellular one was measured from cell lysate. Results refer to the percentage of extracellular LDH on total LDH (where total is calculated as extracellular + intracellular content).

### Bradford assay

Bradford protein assay (Quick Start™ Bradford Protein Assay Kit, Biorad, Hercules, CA, USA) was performed to quantize protein levels. Cells were treated and lysed in 100 μL Triton X-100 (0,9%) diluted at a ratio of 1:10. In total, 10 μL of each treatment was used to calculate the amount of protein per well. BSA was chosen as standard to develop a calibration curve ranged from 1 mg/mL to 0 mg/mL and the protein amount was measured as a function of absorbance at 570 nm using a microplate photometer (Victor 4, PerkinElmer, Waltham, MA, USA). Results were expressed as a total μg of proteins per well. Bradford assay was performed at 24 h, 48 h, 72 h.

### BrdU proliferation assay

Cell proliferation was measured by bromodeoxyuridine (BrdU) incorporation using the BrdU Cell Proliferation Assay Kit purchased from Cell Signaling (Cat. No.:6813). Cells were plated at a density of 10,000 cells/well in 96-well plates. BrdU was added overnight during the 16–20 h prior to the end of the incubation. The assay was performed according to the manufacturer's instructions. The kit detects 5-bromo-2'-deoxyuridine (BrdU) incorporated into cellular DNA during cell proliferation using an anti-BrdU antibody. When cells are cultured in a medium containing BrdU, this thymidine analogue is incorporated into the newly synthesized DNA of proliferating cells. After removing the medium, the cells are fixed, and the DNA is denatured using a fixation/denaturation solution. A mouse monoclonal anti-BrdU antibody is then added to detect the incorporated BrdU (DNA denaturation is necessary to improve the accessibility of the incorporated BrdU to the detection antibody). The anti-mouse IgG-HRP conjugated antibody is then used to recognize the bound detection antibody. The HRP substrate (IMB) is added to detect the color. The absorbance of the developed color is proportional to the amount of BrdU incorporated into the cells, which is a direct indication of cell proliferation. The obtained data is represented in a graph showing the percentage (%) of BrdU measured relative to the control for each tested dose.

### Western immunoblot

Both U87MG and T98G were plated in T25 flask at a density of 500.000 cells and treated at 4 h and 24 h**.** After 4 h or 24 h, cells were scraped in PBS without Ca^2+^ and Mg^2+^ and centrifuged at 1100 rpm for 5 min. Then, cells were lysed in RIPA buffer [1 mM EDTA (Cat. No.: E7889), 150 mM NaCl (Cat. No.: S9888), 1% igepal (Cat. No.: I3021), 0.5% sodium deoxycholate (Cat. No.: D-6750), 50 mM Tris–HCl, pH 8.0 (Cat.No.: T-3038) (Sigma-Aldrich, St. Louis, MO, USA), and 0.1% sodium dodecyl sulphate, SDS, (Cat. No.:1610416—Bio-Rad, Hercules, CA, USA)] containing protease inhibitor cocktail diluted 1:250 (Cat. No.: P8340—Sigma–Aldrich, St. Louis, MO, USA). The total protein amount was measured following the Bradford protein assay, as described above. Precast BIS–TRIS polyacrylamide gels with a 4–12% polyacrylamide gradient (Invitrogen, Carlsbad, CA, USA) were used for the experiments. 30 μg of proteins/sample were mixed with 4 × Bolt™ LDS Sample Buffer (Cat. No.: B0007—Novex, Carlsbad, CA, USA) and 10 × Bolt™ Sample Reducing Agent (Cat. No.: B0009—Novex, Carlsbad, CA, USA), boiled for 5 min at 95 °C and, eventually, undergone to the electrophoresis. Then, proteins were transferred to a PVDF membrane (Invitrogen, Carlsbad, CA, USA) using iBlot™ 2 Gel Transfer Device (Invitrogen, Carlsbad, CA, USA) and different antibodies were tested. Primary antibodies were all prepared in Flex Solution (iBind™ Flex Solution Kit, Invitrogen, Carlsbad, CA, USA) and were incubated overnight at 4 °C with gentle shaking; except for β-actin and pCREB antibodies, which were incubated for 2 h. The day after, the primary antibody was removed, and the membrane was washed three times with TBS-T. After that, the membrane was incubated for 1 h with the secondary antibody, dissolved in Flex Solution (iBind™ Flex Solution Kit, Invitrogen, Carlsbad, CA, USA). Following three washes in TBST, bands were detected by chemiluminescence (ChemiDoc™ XRS, Biorad, Hercules, CA, USA), rinsing the membrane with ECL reagents (SuperSignal™ West Pico PLUS Chemiluminescent Substrate, Thermo Scientific™, Rockford, IL, USA, and Pierce™ ECL Western Blotting Substrate).

Antibody information used for Western immunoblotting analysis:

**Table:** 

Antibody	Diluition	Producer	Catalog Number
*β-actina*	*1:1000*	*Sigma-Aldrich*	*#A 5316*
*pCREB (ser133)*	*1:1000*	*ThermoFisher*	*PA1-4619*
*Total CREB*	*1:500*	*ThermoFisher*	*PA1-850*
*P21*	*1:500*	*Cell signaling*	*#64016*
*LC3A*	*1:250*	*Novus Biologicals*	*NB100-2331*
*Beclin 1*	*1:1000*	*Novus Biologicals*	*NB500-249*
*Cleaved caspase 3 (Asp 175)*	*1:1000*	*Cell signaling*	*#9661*
*pSTAT 3*	*1:500*	*Cell signaling*	*#9145*
*totSTAT 3*	*1:1000*	*Cell signaling*	
*NFKB*	*1:1000*	*Cell signaling*	*#8242*
*GAPDH*	*1:1000*	*Cell signaling*	*#2118*
*Anti-mouse*	*1:3000*	*Sigma-Aldrich*	*#A 3682*
*Anti-rabbit*	*1:15000*	*JacksonImmuno*	*#111–035–045*

### IL-6 assay

According to manufacturer's instructions, the Human Interleuchin 6 (IL 6) levels in the culture media were assayed by enzyme-linked immunosorbent assay (Human IL-6 DuoSet ELISA, Cat No: DY206-05, R&D Systems). Cells were plated at a density of 10,000 cells/well in 96-well plates. The day before the dosage, the wells were coated with capture antibody overnight. At the end of treatment, the culture medium was collected and then diluted 1:2 to quantify interleukin levels and the other steps were performed as instructed. The results were calculated, through a calibration curve in the range 9.4—600 pg/mL, as picograms of IL 6 normalised to milligrams of total proteins.

### RNA extraction for RT-PCR and real-time PCR

Total cytoplasmic RNA was extracted using the RNeasy Micro Kit (Qiagen, Hilden, Germany), which included a 15-min DNase treatment. RNA concentration was measured using the Qubit™ RNA HS Assay Kit (Thermo Fisher Scientific). Aliquots of RNA were reverse transcribed into cDNA using random hexamer primers.

Quantitative changes in mRNA levels were assessed by real-time PCR using the following cycling conditions: one denaturation cycle at 95 °C for 3 min; 40 amplification cycles at 95 °C for 5 s and 60 °C for 10 s; followed by one melting cycle at 95 °C for 1 min, 60 °C for 30 s, and 95 °C for 30 s, using the Brilliant III Ultra-Fast SYBR® Green QPCR Master Mix (Stratagene, La Jolla, CA, USA). PCR reactions were performed in a final reaction volume of 20 µL using the AriaMx real-time PCR system (Stratagene).

The primers used for gene expression analysis targeted P2X7 receptor (P2X7R) isoform A, B and Bs. Relative mRNA concentrations were calculated from the take-off point of the amplification curves (threshold cycle, Ct) using the comparative quantification method provided by the Stratagene software and based on the 2^ − ΔΔCt method.

This analysis estimates the target mRNA level of a given sample relative to the mean target mRNA levels of untreated control samples (calibrator value), thereby allowing statistical analysis of deviations from the mean, including among control samples. Ct values for GAPDH expression were used as the normalization signal. In each assay, PCR efficiency was also calculated using serial dilutions of an experimental sample.

### Statistical analyses

Each experiment was repeated at least three times. All the statistical analyses were performed with PrismTM computer program (GraphPad, San Diego, CA, USA Software version 7.4). Data were analyzed by a one-way ANOVA, followed by Dunnett’s test. Statistical significance was determined at α = 0.05 level. Differences were considered statistically significant when p < 0.05.

## Results

### Determination of P2X7R isoform A and B expression in U87MG and T98G GBM cell cultures

**Fig. 1:**
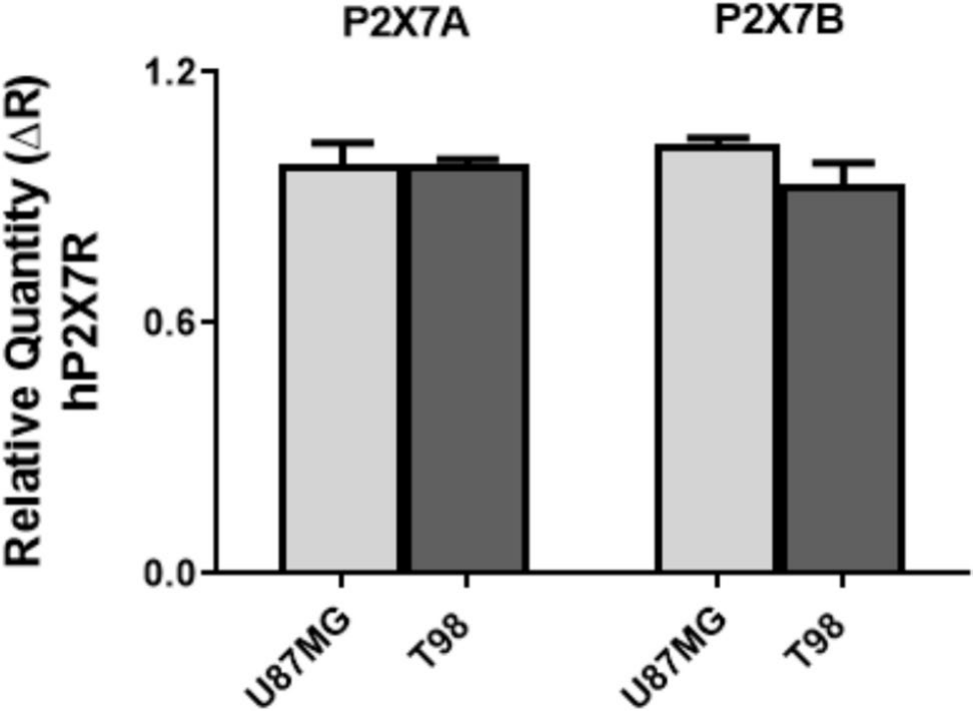
P2X7R isoforms A and B expressions in U87MG and T98 GBM cell cultures. Both P2X7A and P2X7B mRNA isoforms are expressed in U87MG and T98 GBM cells. In U87MG cells, equal expressions of isoforms A and B are observed, while in T98 cells, slightly lower expression of the B isoform is observed compared to the A isoform

We evalueted P2X7R A and B mRNA levels in U87MG and T98 cells using real-time PCR. In U87MG cells, we measured equal levels of expression of isoforms A and B, while in T98 cells the expression levels of the B isoform were lower than those measured in U87MG cell cultures. Furthermore, in T98 cells the levels of the A isoform were slightly higher than those of the B isoform (Fig. 1).

### Effects of P2X7R activation/inhibition in U87MG and T98G cell cultures

**Fig. 2:**
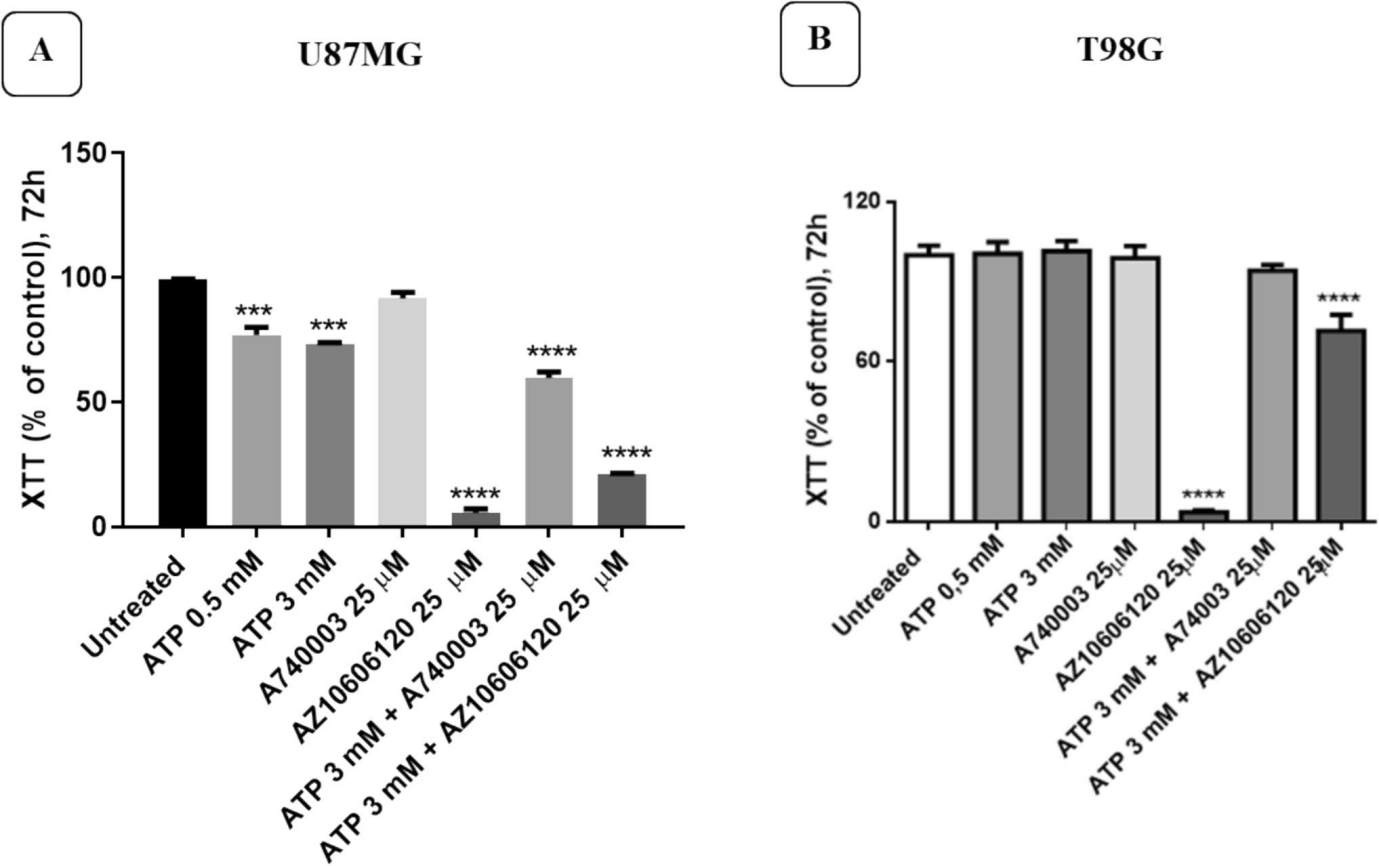
Cell viability of U87MG and T98G GBM cultures after 72 h of treatment with ATP, A740003, and AZ10606120. The figure shows the effects of ATP (0.5 and 3 mM), the antagonists A740003 (25 µM) and AZ10606120 (25 µM), or the combination of ATP (3 mM) plus AZ10606120 (25 µM) or A740003 (25 µM), on U87MG (**A**) and T98G (**B**) viability after 72 h of treatment. Cell viability was expressed as a % of the control. Values represent the mean ± standard error (SEM) of three independent experiments performed in six replicates and evaluated by one-way ANOVA followed by Dunnett's post-test. (*N* = 3) **p < 0.01; ***p < 0.005; ****p < 0.0001. All *p*-values ​​were calculated relative to the control

U87MG and T98 cells were treated for 72 h with (0.5 and 3 mM) ATP, with 25 µM A740003 and 25 µM AZ10606120 and finally with the association 3 mM ATP plus 25 µM AZ10606120 or 3 mM ATP plus 25 µM A740003 (Fig. 2A and B). ATP did not alter T98G cellular activity, whereas a slight reduction was shown in U87MG. Antagonist A740003 also had no effect, while AZ10606120 alone significantly reduced cell viability. When ATP and AZ10606120 were incubated together in cell cultures, 3 mM ATP partially restores cell growth especially in T98 cells.

### Effects of the P2X7R antagonist AZ10606120 on cell viability, cytotoxicity, and cell proliferation

**Fig. 3:**
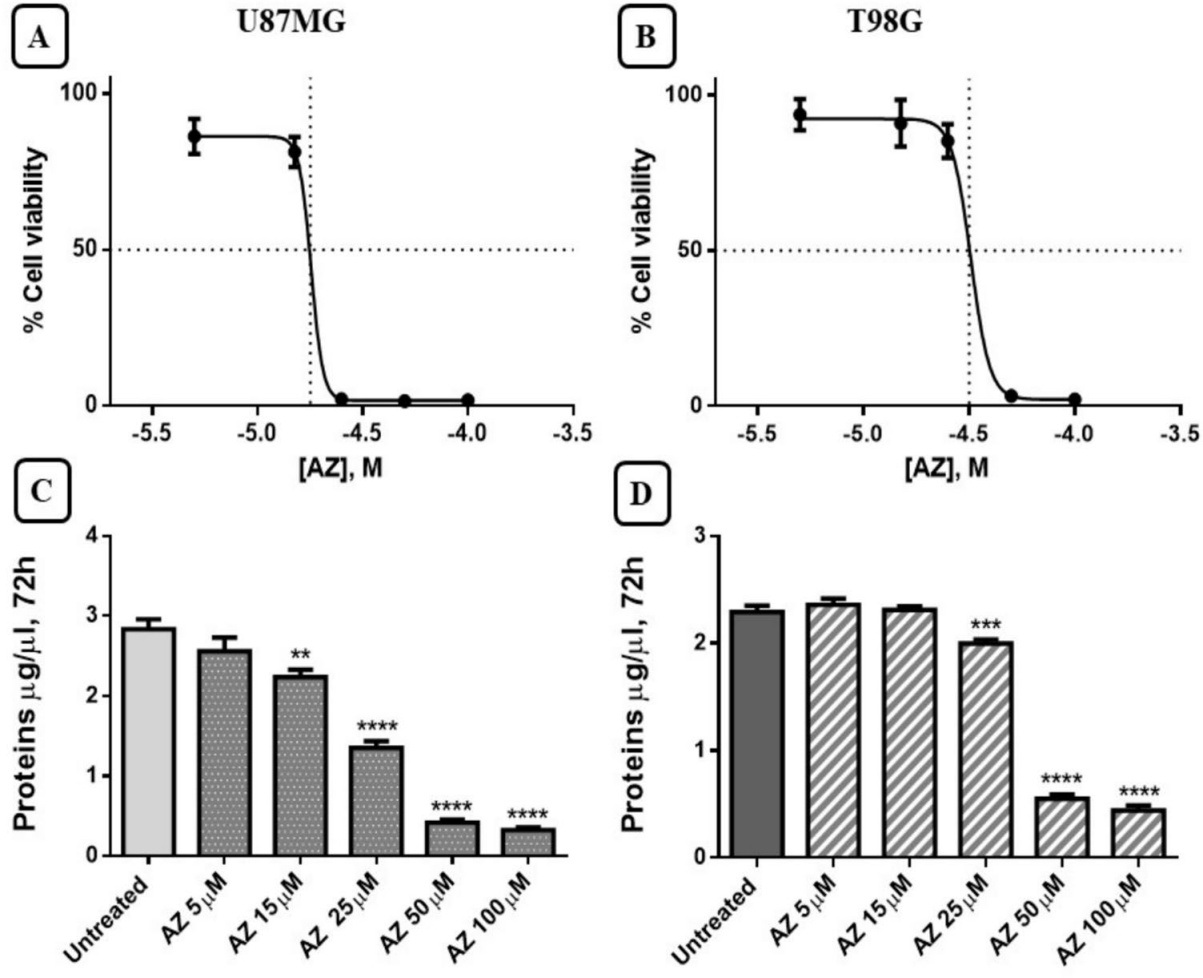
Cell viability and protein measured in U87MG and T98G GBM cells after treatment with AZ10606120. The effects of the antagonist AZ10606120 (5–100 µM) were assessed using the XTT assay (Fig. 3**A** and **B**). Cell viability was expressed as % of the control. Figures 3**C** and **D** show protein (µg/µl) measured by the Bradford method after 72 h of treatment with AZ10606120 (5–100 µM). Values represent the mean ± standard error (SEM) of three independent experiments performed in six replicates and evaluated by one-way ANOVA followed by Dunnett's post-test. (N = 3) **p < 0.01; ***p < 0.005; ****p < 0.0001. All *p*-values were calculated relative to control

As shown in Figs. 3 A and B, 72-h exposure to various concentrations of AZ10606120 (5–100 µM) reduced cell viability as assessed by the XTT assay. At the 15 µM concentration, a reduction in cell growth of approximately 20 ± 1.05% was observed in the U87MG cell line, while at the same concentration, cell viability was reduced by approximately 10 ± 0.50% in T98G cells. At the highest concentrations (50 and 100 µM), no viable cells were detected in the two cell lines treated with AZ10606120. Dose–response analysis quantified an IC50 of 17 ± 0.85 and 31 ± 1.55 µM for AZ10606120 in the U87MG and T98G cell lines, respectively (Fig. 3A and B). Figures 3C and D illustrate the effect of 72 h of exposure to AZ10606120 (5–100 µM) on total protein measured by the Bradford method. As shown in the graph, protein levels decreased significantly at the highest concentrations, 25, 50, and 100 µM, in both the U87MG and T98G cell lines (Fig. 3C and D).

**Fig. 4:**
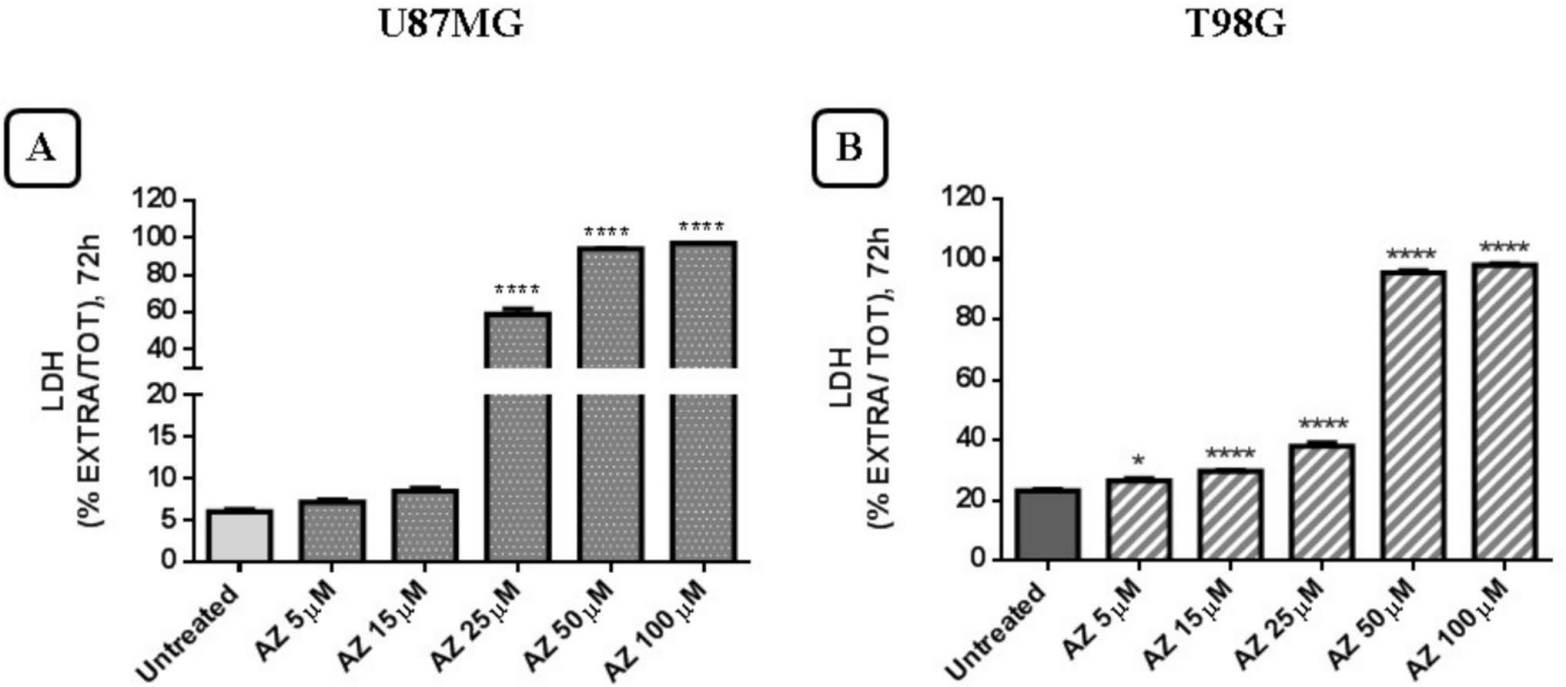
Evaluation of the amount of LDH released after treatment with AZ10606120 in U87MG (**A**) and T98G (**B**) cell lines. The figures show the concentrations of AZ10606120 versus the ratio between the amount of LDH released and the total amount (total amount was calculated as extracellular + intracellular LDH content). The values represent the mean ± standard error (SEM) of three independent experiments performed in six replicates and evaluated by one-way ANOVA followed by Dunnett's post-test. (*N* = 3) *p < 0.05; **p < 0.01; ***p < 0.005; **** *p* < 0.0001. All *p* values ​​were calculated with respect to the control

The assay measuring LDH released from damaged cells can be used to monitor cytotoxicity in different cellular models. After 72 h of exposure (5–100 µM) with AZ10606120, a percentage increase in extracellular LDH compared to total LDH was observed. Both cell lines analyzed showed similar behavior with some quantitative differences. The U87MG culture showed greater sensitivity to higher concentrations (25, 50, 100 µM) of AZ10606120 compared to the T98G cell line (Fig. 4A and B).

**Fig. 5:**
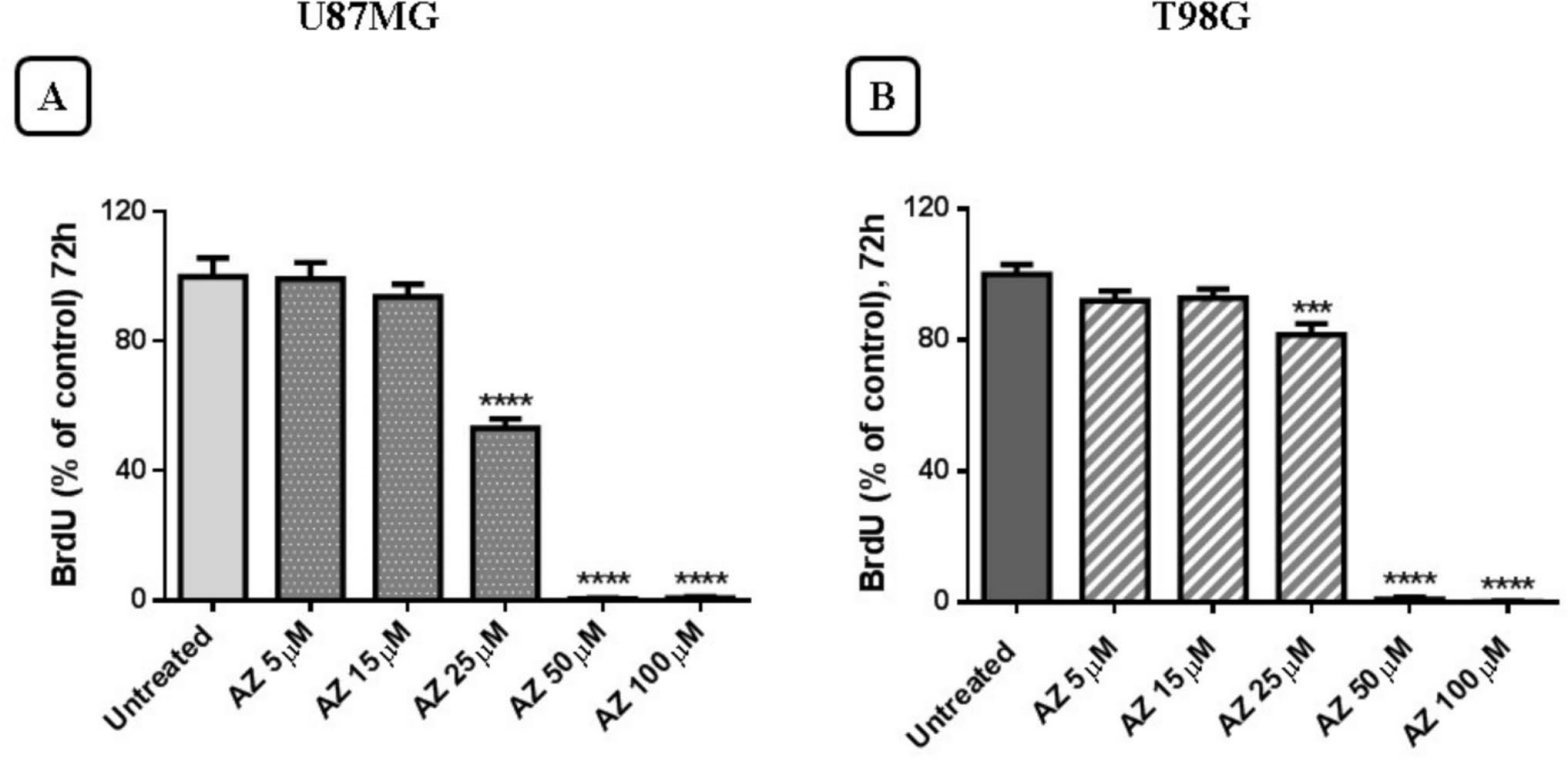
Newly synthesized cells are assessed by the BrdU proliferation assay after 72 h of treatment with AZ10606120 (5–100 µM). Panel **A** show results in U87MG cell line and Panel **B** show results in T98G cell line. Data are expressed as the percentage of AZ10606120-treated cells capable of incorporating BrdU compared to untreated control cells. Values ​​represent the mean ± standard error (SEM) of three independent experiments performed in six replicates and evaluated by one-way ANOVA followed by Dunnett's post-test. (*N* = 3) ***p < 0.005; ****p < 0.0001. All p-values ​​were calculated relative to the control

Proliferation of newly synthesized cells was assessed by the BrdU incorporation assay after treatment with AZ10606120 (5–100 µM) for 72 h. Since cell proliferation requires DNA synthesis, one method to measure it is to monitor the uptake of the modified thymidine nucleotide BrdU. The assay used is based on the incorporation of BrdU into the newly synthesized DNA strands of proliferating cells. Both cell lines used confirm that proliferating cells are reduced following treatment with AZ10606120. Furthermore, in this assay, the U87MG cell line showed greater sensitivity than the T98G line; a significant reduction in cell growth of the U87MG line was observed at a concentration of 25 µM of AZ10606120 (Fig. 5A and B).

### Effects of P2X7R blockade by AZ10606120 on p21 and cleaved caspase-3 gene expression

To investigate the molecular mechanisms underlying cellular toxicity caused by AZ10606120 in U87MG and T98G cell lines, we analyzed several key markers involved in apoptosis and cell survival. We measured p21 and cleaved caspase-3 protein expression in U87MG and T98G cells treated with AZ10606120 (5, 15, and 25 µM) and in untreated control cells. To perform these experiments, we used the lowest concentrations proven effective in previous assays, since at the lowest AZ10606120 concentrations, viable cells are still present and can be analyzed for changes in their protein content.

**Fig. 6:**
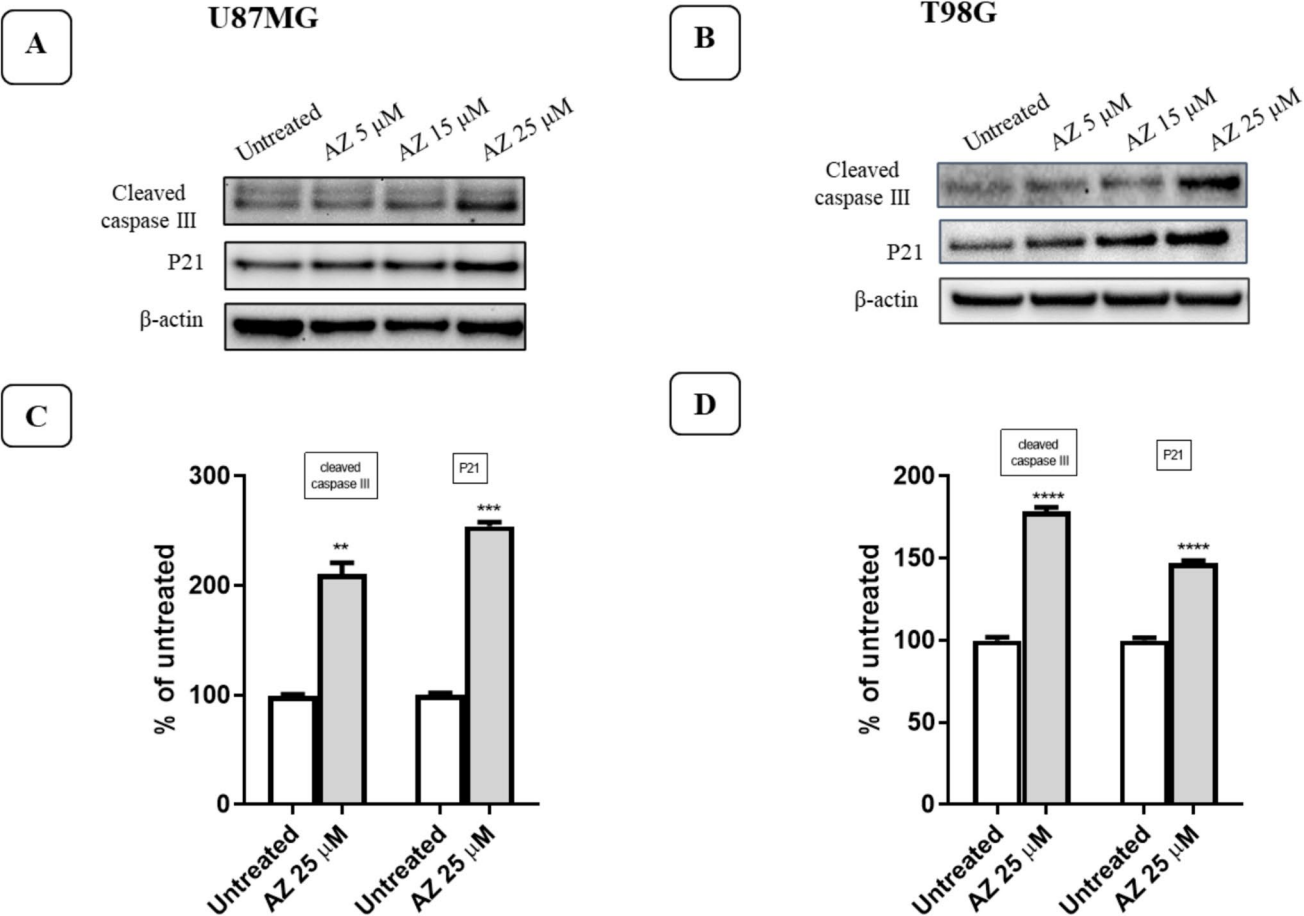
Effects of 24-h treatment with AZ10606120 (5, 15, and 25 µM) on p21 and cleaved caspase-3 expression in U87MG (**A**-**C**) and T98G (**B**-**D**) cell lines

P21, a cyclin kinase inhibitor capable of blocking the cell at any stage of its cycle, showed increased expression in both cell lines analyzed (Fig. 6A and B). As shown in Fig. 6C and D, p21 protein expression increased by 170.14 ± 2.45% and 181.45 ± 2.70% compared to the control, respectively, in the U87MG and T98G cell lines after 24-h treatment with 25 µM AZ10606120. Furthermore, both cell lines showed increased expression of cleaved caspase-3 (Fig. 6A and B). Cleaved caspase-3 is the active form of caspase-3; caspase-3 is an initiator caspase capable of activating other caspases, leading the cell to apoptosis. The expression of cleaved caspase-3 increased by 217.61 ± 18.80% and 236.15 ± 2.45% in U897MG and T98G cell lines, respectively, after inhibition of P2X7R with 25 µM AZ10606120 (Fig. 6C and D).

The expression of p21 and cleaved caspase-3 proteins was determined by Western blotting after treatment with AZ10606120 in the U87MG and T98G cell lines. Figures 6A and B show the expression of p21 and cleaved caspase-3 proteins in the control (lane 1) and in cells treated for 24 h with different concentrations of AZ10606120 (lane 2 = 5 µM; 3 = 15 µM and 4 = 25 µM). Figures 6C and D show the percentage change in expression of the analyzed proteins relative to actin expression (loading control) for the 25 µM concentration of AZ10606120. The mean value was determined by densitometric analysis of Western blots, normalized to actin expression as a loading control (*N* = 3). Values ​​represent the mean ± standard error (SEM) of three independent experiments performed in six replicates and evaluated by one-way ANOVA followed by Dunnett's post-test. (N = 3) *** *p*< 0.005; **** *p*< 0.0001. All *p*-values ​​were calculated relative to the control.

### P2X7R receptor blockade by AZ10606120 in the autophagy pathway

**Fig. 7:**
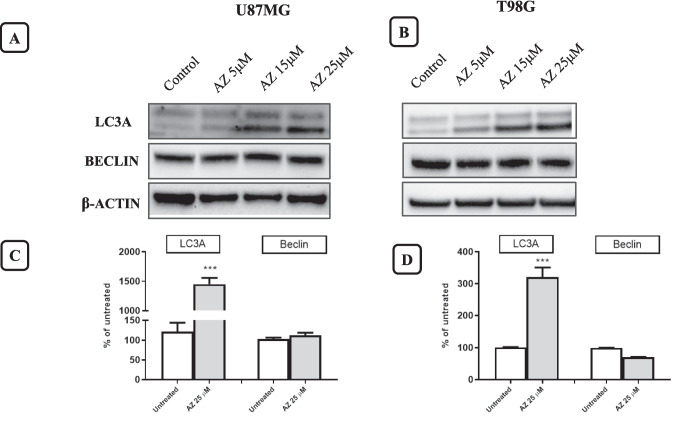
Evaluation of LC3A and beclin-1 activation after treatment with AZ10606120 (5, 15, 25 µM). LC3A and beclin-1 protein expression were determined by Western blotting after treatment with AZ10606120 of the U87MG and T98G cell lines. Figures 7**A** and **B** show the expression of LC3A and beclin-1 proteins in control (lane 1) and in cells treated for 24 h with different concentrations of AZ10606120 (lane 2 = 5 µM; 3 = 15 µM and 4 = 25 µM). Figures 7**C** and **D** show the percentage change in the expression of the analyzed proteins compared to the expression of β-actin (loading control) for the AZ10606120 concentration of 25 µM. The mean value was determined by densitometric analysis of Western blots, normalized to β-actin expression as a loading control (*N* = 3). Values ​​represent the mean ± standard error (SEM) of three independent experiments performed in six replicates and evaluated by one-way ANOVA followed by Dunnett's post-test. (*N* = 3) ***p < 0.005; ****p < 0.0001. All *p*-values ​​were calculated relative to the control

As shown in Figs. 7A and B, treatment of the U87MG and T98G cell lines with AZ10606120 (5, 15, and 25 µM) increased LC3A expression in both cell lines. LC3A is a crucial protein in the autophagy process. In autophagy, the cell, in response to certain types of insults, removes and recycles damaged cellular components. Proteins and organelles are sequestered in a specific cellular region surrounded by a membrane (autophagosome). The resulting vesicle is then fused with a lysosome, and the contents are degraded and reused by the cell. The LC3A protein is involved in autophagosome formation and lysosome fusion, interacts with other proteins, and plays a role in regulating apoptosis and/or modulating other metabolic pathways. Unlike LC3A, beclin-1 protein was not altered by AZ10606120 treatment.

### Effects of ATP and AZ10606120 on IL-6 release

**Fig. 8:**
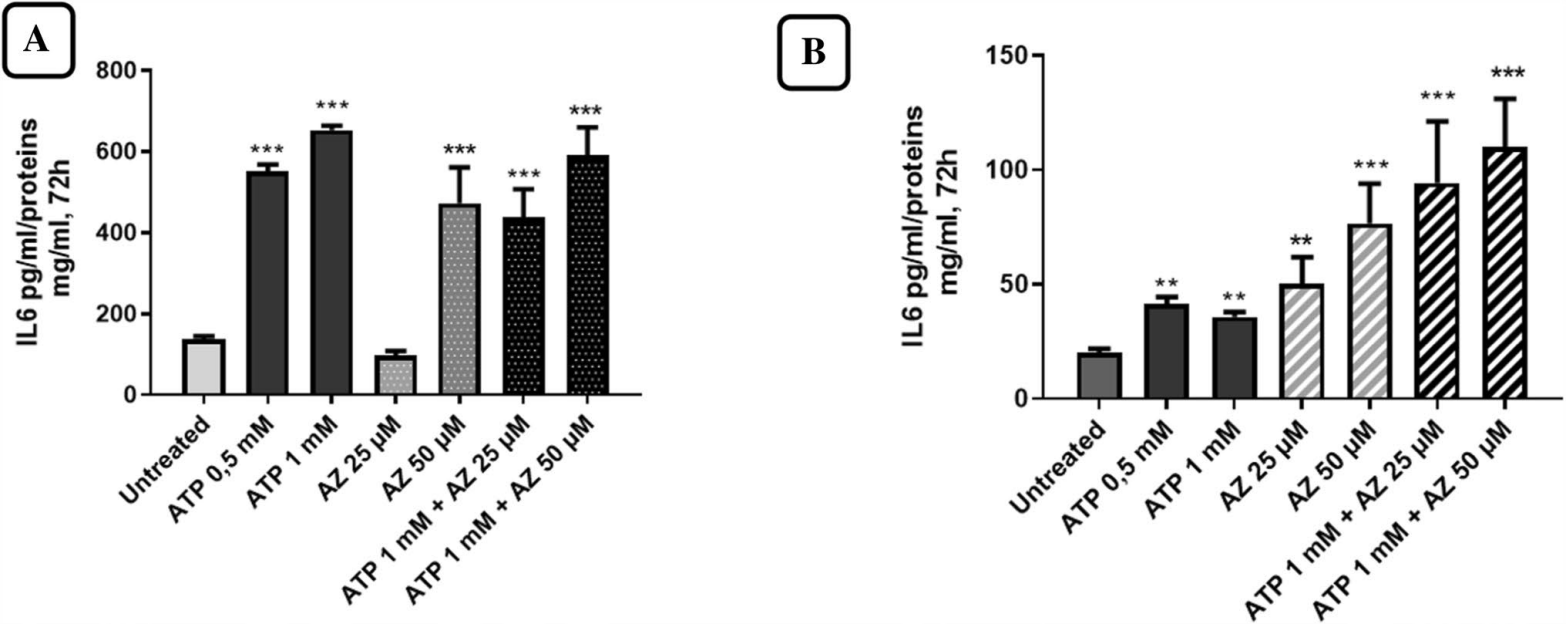
Amount of IL-6 measured in the culture medium after treatment of U87MG (**A**) and T98G (**B**) for 72 h with ATP, AZ10606120, and ATP plus AZ10606120. The amount of IL-6 present in the extracellular culture medium was determined by referring to an IL-6 calibration curve with concentrations ranging from 9.40 to 600 pg/mL, normalizing pg of IL-6 to milligrams of total protein. Values ​​represent the mean ± standard error (SEM) of three independent experiments conducted in triplicate, evaluated by one-way ANOVA followed by Dunnett's post-test. *p < 0.05; **p < 0.01; ***p < 0.005; ****p < 0.0001. All *p* values ​​were calculated relative to the control

IL-6 concentrations in the cultures were measured by ELISA after 72 h of treatment with ATP, AZ10606120, and ATP plus AZ10606120. Extracellular IL-6 levels increased significantly in response to the application of ATP (0.5 and 1 mM) (Fig. 8A and 8). In the presence of 1 mM ATP, an approximately three-fold increase in the amount of IL-6 released into the culture medium was observed compared to baseline values. The antagonist AZ10606120, used at concentrations of 25 and 50 µM, was unable to reverse the stimulatory effect of ATP on basal IL-6 release (Fig. 8A and B). Surprisingly, the antagonist AZ10606120 himself stimulated IL-6 release. The effect of AZ10606120 was significant at a concentration of 25 and 50 µM. We did not use higher concentrations of AZ10606120 to avoid reducing the number of viable cells. These effects were observed in both cell lines. In these experiments, the U87MG cell line also showed greater sensitivity to the effects of ATP and AZ10606120 than the T98G cell line. Experiments conducted with the T98G cell line showed greater variability, but high significance was still observed.

### Effects of treatment with ATP, AZ10606120 and of the association ATP plus AZ10606120 on the expression of the transcription factors CREB, STAT, and NF-κB

**Fig. 9:**
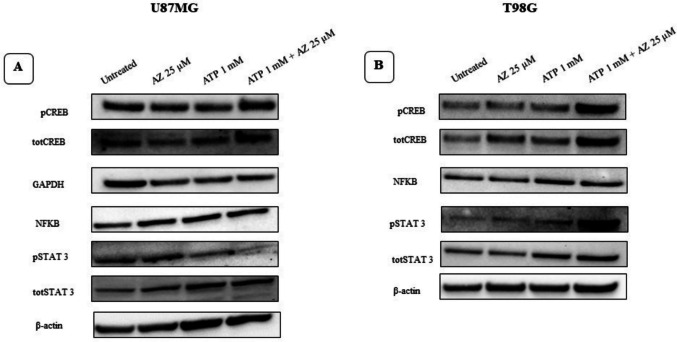
In U87MG (**A**) and T98G (**B**) cells, we measured the expression of CREB, STAT3, and NF-κB after treatment with ATP (1 mM), AZ10606120 (25 µM) and ATP (1 mM) plus AZ10606120 (25 µM). The data refer to the following treatments: lane 1: untreated cells; lane 2: cells treated with 25 µM AZ10606120; lane 3: cells treated with 1 mM ATP and lane 4: cells treated with 1 mM ATP plus 25 µM AZ10606120 for 4 h, in U87 and T98, respectively. For each protein set, GAPDH or β-actin are reported as the normalizer gene

In the experiments shown in Figs. 9A and B, after exposure to 1 mM ATP, 25 µM AZ10606120, and 1 mM ATP plus the antagonist AZ10606120, we measured the expression of the transcription factors CREB, STAT3 and NF-κB by Western blot analysis. Treatments with 1 mM ATP and exposure to 25 µM AZ10606120 stimulated increases in the levels of phosphorylated CREB (pCREB) and total CREB (totCREB), phosphorylated STAT3 (pSTAT3), and total STAT3 (totSTAT3) in the two cell lines analyzed. AZ10606120 (25 µM) did not reverse the effects of ATP. Exposure to ATP and AZ10606120 did not alter NF-κB expression in both cell lines.

## Discussion

Current literature reflects contradictory findings on P2X7R activity in GBM. P2X7R is known to be expressed in both glial cells [26, 27] and tumor-infiltrating microglia [28], therefore, it is reasonable to expect that P2X7R-mediated effects occur in both cell types. Furthermore, the expression of ATP-hydrolyzing enzymes localized in the tumor microenvironment (TME) has been shown to be reduced, resulting in prolongation of ATP effects and increased likelihood of P2X7R stimulation in GBM cells [29]. In *vitro* studies have shown that P2X7R inhibition promotes cell growth in rat glioma C6 [30] and mouse GL261 [24] cells, suggesting a cytolytic activity of P2X7R. Conversely, other studies have observed that P2X7R inhibition reduces cell growth in glioma [31–33], an effect indicative of a basal trophic activity of P2X7R. These differences can probably be explained by the heterogeneity of the glioma models used (cell lines isolated from mice, rats and humans), since each cell line has its own characteristics and a different level of P2X7R expression [34].

The diversity of effects observed on cell growth produced by P2X7R activation/inhibition may also be related to the presence of variants of the P2X7R receptor. Among the various isoforms that can be generated by alternative splicing of the human P2X7R gene, isoforms A and B give rise to well-characterized and ubiquitously expressed functional ion channels [35]. While P2X7A corresponds to the full-length variant, P2X7B results from the retention of an intron containing a stop codon, which shortens the length of the protein [36, 37]. This truncated version lacks the C-terminal tail that is crucial for macropore opening. The ability to form the macropore is responsible for the cytotoxic activity of P2X7R. The P2X7B variant is functional and retains the ability to open the ion channel, resulting in its own cellular effects [37, 38]. Several studies have shown that the P2X7A variant has cytotoxic and antitumor effects, while the P2X7B variant displays trophic and metastatic properties on tumor cells. The antagonists available to date do not show selectivity for one form of P2X7R or the other.

To clarify the effects of P2X7R on cell growth in GBM, we measured the expression of the two A and B variants in the GBM cell cultures used in our study, the U87MG and T98G cell lines. In our experiments, we observed that both A and B isoforms are present in U87MG and T98G cells, with a slightly higher expression of the P2X7B variant in the U87MG cell line. To evaluate the effects of P2X7A and P2X7B on cell proliferation capacity, U87MG and T98G cultures were exposed to ATP and two different antagonists, the compounds AZ10606120 and A740003 (a lipophilic brain-permeant compound).

Exposure of cell cultures to different concentrations of ATP did not produce any significant changes in cellular activity in U87MG and T98G glioma cultures. However, 72-h exposure to various concentrations of the antagonist AZ10606120 significantly reduced cell viability and proliferation. This tumor growth-inhibiting effect observed with the antagonist AZ10606120 was not observable after treatment with the compound A740003. The lack of an effect on cell growth in U87MG and T98G cultures by ATP may be explained by the fact that ATP concentrations in the TME are already very high and can produce basal activation of P2X7R. The P2X7 receptor does not desensitize, remains active in the presence of an agonist, and closes when the agonist is removed. Furthermore, cells expressing the P2X7 receptor release a greater amount of ATP than that released by cells that do not express it [39] and furthermore ATP is less metabolized due to the reduced enzymatic activity present in the TME [29].

In contrast, inhibition of P2X7R with the antagonist AZ10606120 produced a significant reduction in cell viability and proliferation, consistent with what was observed in various experimental assays. The XTT cell viability assay demonstrated that AZ10606120 significantly reduced cell viability in both U87MG and T98G cell lines. At the same concentrations, AZ10606120 caused an increase in the amount of extracellular LDH compared to the total amount present in both cell lines analyzed. In the BrdU incorporation assay, AZ10606120 effectively reduced BrdU incorporation into cells.

Treatment with AZ10606120 significantly reduced the number of tumor cells in both U87GM and T98G cells, although a higher sensitivity to AZ10606120 was observed in the U87GM cell line (especially detectable at the lowest concentrations of AZ10606120 used), compared to the T98G cell line. The results obtained therefore appear similar in the two cell lines used with small quantitative differences that may be due to a higher expression of the B isoform (provided with cellular proliferative activity) present in U87MG cells compared to T98 cells. Our results overall suggest that the compound AZ10606120 can block cell growth and proliferation; the antitumor activity of this compound has also been widely reported by other authors [33, 40].

Unlike the compound AZ10606120, the antagonist A740003 showed no effect on tumor growth. The antagonists AZ10606120 and A740003 act as negative allosteric modulators. Although binding to the allosteric pocket of the receptor is common to these compounds, there are specific differences in their binding modes and interactions. AZ10606120 interacts with a more superficial site of the allosteric pocket than the deeper site bound by A740003. This diversity of binding was also confirmed by the fact that mutations at the entrance of the binding pocket have a greater impact on the sensitivity of the receptor to AZ10606120 than to A740003 [41].

These differences could have a major influence on binding to a P2X7 receptor that could be probably composed of isoforms A and B. In the U87MG and T98 cells used, isoforms A and B are expressed in similar amounts. These isoforms could co-assemble to form a P2X7 receptor that is more sensitive to the antagonist AZ10606120, which binds the receptor at a more superficial site.

To understand the molecular mechanisms underlying the antitumor effects observed with AZ10606120 treatment, we assessed the expression of key proteins involved in the cell cycle, apoptosis, and autophagy using Western blot experiments. In these experiments, we used the lowest active concentrations of AZ10606120 to avoid completely suppressing cell viability. p21 is a key regulatory protein in the cell cycle, known for its ability to block cell proliferation after exposure to DNA-damaging agents. Exposure to AZ10606120 for 24 h induced an increase in p21 expression in both cell lines used. The expression of cleaved caspase-3, the active form of caspase-3, was also assessed. Cleaved caspase-3 is a key factor in the execution phase of cellular apoptosis. In both cell lines analyzed we observed a clear increase in the expression of cleaved caspase-3 following exposure of cell cultures to AZ10606120. Activation of caspase-3 causes specific changes in the morphology and biochemistry of apoptotic cells; such changes are considered hallmarks of apoptosis [42]. In other experiments, we evaluated the expression of LC3A and beclin-1 proteins, two proteins involved in the regulation of autophagy. Autophagy is a process that maintains cellular homeostasis in response to cellular damage [43].

The increased expression of LC3A, p21 and cleaved caspase-3 proteins involved in cell survival mechanisms, observed after P2X7R blockade with AZ10606120, demonstrates that P2X7R silencing activates repair and cell survival mechanisms that can induce autophagy and cell apoptosis [44, 45]. P2X7R therefore appears to have a trophic role in human GBM U87MG and T98 cells that is inhibited by treatment with the antagonist AZ10606120.

Finally, we aimed to evaluate the effects of AZ10606120 on the stimulation of ATP-induced IL-6 release in U87MG and T98G cell lines. Glioma cells release inflammatory-stimulating cytokines that facilitate cell proliferation [46], and activation of P2X7R by ATP can stimulate the release of several inflammatory cytokines such as interleukin-1β (Il-1β) and IL-8 [28, 46]. IL-6 is a cytokine known to be important in the activation of several pro-oncogenic signaling pathways in glioblastoma [25]. IL-6 has become a key target in oncology research due to its essential roles in inflammation, angiogenesis, and immune response [47]. Elevated levels of IL-6 have been consistently associated with a poor prognosis in various cancer types and in GBM [48, 49]. IL-6 promotes among other things the formation of peritumoral edema. The presence of peritumoral edema is associated with more difficult surgical removal, a higher risk of postoperative complications and a shorter survival in patients with gliomas and meningiomas [50].

In our experiments, we observed that exposure of U87MG and T98G cell cultures to ATP for 72 h stimulated IL-6 release in a concentration-dependent manner. The effect of ATP was not reversed by the antagonist AZ10606120; indeed, unexpectedly, treatment with the antagonist alone was able to stimulate IL-6 release. These results may be explained by the fact that P2X7R inhibition by AZ10606120 may activate alternative signaling pathways that promote IL-6 release as part of an adaptive response of the cell to counteract the antitumor effect of AZ10606120. IL-6 primarily activates the JAK-STAT signaling pathway, leading to the activation of the transcription factor STAT3. This factor dimerizes and translocates to the nucleus to induce the expression of pro-inflammatory and survival genes.

Furthermore, ATP or its metabolites present in the extracellular environment can activate purinergic receptors other than the P2X7 receptor (in fact the effect of ATP was not reverted by AZ10606120 but rather the effects of the two compounds seem to be additive), which are able to trigger pro-inflammatory pathways, such as the activation of NF-κB, CREB and STAT3 [51]. Based on these considerations, in another series of experiments, we measured the expression of STAT3, CREB and NF-κB in response to short-term (4 h) treatment with ATP and AZ10606120 to evaluate the possible effects of these compounds on the signaling pathways activated by the IL-6 receptor.

STAT3 is a key protein that acts as a transcription factor, regulating genes involved in cell growth, survival, differentiation and immune response. CREB is a transcription factor regulated by phosphorylation following the activation of various signaling pathways [52]. Studies conducted in cancer patients in animal models and cell lines have demonstrated that CREB is implicated in cancer signaling pathways [53]. Our results indicate that treatment of glioma cells with ATP and AZ10606120 stimulates an early increase in the expression of STAT3 (total and phosphorylated) and CREB protein (total and phosphorylated), in U87MG and T98G cells. While NFKB expression was not modified. These data, as well as previous data observed on IL-6 release, indicate that P2X7R inhibition can activate different receptor pathways in response to extracellular ATP. ATP is a cellular paracrine regulatory signal that acts by activating receptors coupled to different intracellular signaling pathways [13] therefore its influence on cellular activity is very broad and can be modified in tumor cells.

This study highlights the potential of P2X7R inhibition as a therapeutic strategy for GBM treatment. Blocking P2X7R inhibits tumor growth and demonstrates that AZ10606120 could be a useful therapeutic approach in the treatment of an aggressive tumor such as GBM. However, it should be considered that treatment with AZ10606120 may promote the onset of resistance mechanisms and trigger the activation of other cellular signaling pathways in response to ATP present in the TME. ATP and its metabolites can accumulate at high levels in the TME and influence cancer growth and progression through various receptors that regulate cellular processes. Increased IL-6 secretion and activation of the CREB and STAT3 pathways are negative prognostic signs that highlight the need to combine P2X7R inhibition with AZ10606120 with other therapies aimed at inhibiting other signaling pathways activated by the cancer cell to obtain a remarkable therapeutic effect.

## Data Availability

Data is provided within the manuscript.
